# Understanding the Process of Drug Addiction Recovery Through First-Hand Experiences: A Qualitative Study in the Netherlands Using Lifeline Interviews

**DOI:** 10.1177/10497323231174161

**Published:** 2023-06-06

**Authors:** T. F. Martinelli, D. P. K. Roeg, L. Bellaert, D. Van de Mheen, G.E. Nagelhout

**Affiliations:** 1 26151 IVO Research Institute, Den Haag, Netherlands; 2 Tranzo Scientific Centre for Care and Wellbeing, School of Social and Behavioural Sciences, 200730 Tilburg University, Tilburg, Netherlands; 3 Kwintes Housing and Rehabilitation Services, Zeist, Netherlands; 4 Department of Special Needs Education, 26656 Ghent University, Ghent, Belgium; 5 Department of Health Promotion, Maastricht University (CAPHRI), Maastricht, Netherlands

**Keywords:** drug addiction, recovery, qualitative study, first-hand experience

## Abstract

Understandings of drug addiction recovery are still being debated. Research on perspectives from first-hand experiences with recovery is rare and often contains short-term experiences in the context of a treatment setting. We aim to gain further understanding of recovery by analyzing autobiographical data from persons in different stages of drug addiction recovery who are not linked to any specific treatment service. We conducted 30 in-depth qualitative interviews with participants from various parts of the Netherlands. Participants self-identified as being “in recovery” or “recovered” from drug addiction for at least 3 months. Men and women are equally represented, and the sample consists of an equal number of participants in early (<1 year, *n* = 10), sustained (1–5 years, *n* = 10), and stable (>5 years, *n* = 10) recovery. We undertook a data-driven thematic analysis. Participants described that recovery is a broad process of change because addiction is interwoven with everything (theme 1); that recovery is reconsidering identity, seeing things in a new light (theme 2); that recovery is a staged long-term process (theme 3); and that universal life processes are part of recovery (theme 4). Thus, Drug addiction recovery is experienced as an interwoven long-term process, including identity change and common or universal life processes. Policy and clinical practice should therefore be aimed at supporting long-term tailored recovery goals and disseminating first-hand recovery experiences to enhance long-term outcomes and reduce stigmatization.

## Introduction

Drug addiction is often described as a chronic relapsing disorder (McLellan et al., 2000; NIDA, 2020). However, evidence shows that most individuals with drug addiction eventually recover (Kelly et al., 2017; White, 2012). The question of what recovery entails is still debated in research, policy, and practice. In Alcoholics Anonymous (AA) literature or studies on how recovery can be achieved and maintained, it is often synonymous with reduction of or abstinence from substance use (Helm, 2019; Kelly & Hoeppner, 2015; Laudet, 2008; Roy et al., 2022). However, a more holistic concept of addiction recovery, inspired by the mental health field (Anthony, 1993; Davidson & White, 2007; Deegan, 1988), has been gaining ground in the addiction field in the last two decades. The latter concept describes recovery as a unique and socially negotiated process (instead of an outcome), characterized by improvements on a variety of personal, functional, and societal life domains (Bathish et al., 2017; Kaskutas et al., 2014; Laudet, 2007; van der Stel, 2013). However, an exact and widely agreed-upon definition of addiction recovery is still lacking (Best & Hennessy, 2022).

Thus far, quantitative studies captured different aspects of recovery and helped us understand that recovery can be a long-term process of several years with subsequent stages (Best et al., 2018; Dennis et al., 2014; Hibbert & Best, 2011; Hser et al., 2007; Martinelli et al., 2020; Simpson & Joe, 2004; Vaillant, 2003), in which growth can be associated with a range of outcomes over time. Recovery capital, for example, is a theoretical construct that attempts to operationalize and capture recovery resources and gains (Best et al., 2012, 2020; Cloud & Granfield, 2008; Groshkova et al., 2013; Hennessy, 2017; Laudet & White, 2008). Researchers also identified mechanisms through which treatment and support contribute to recovery (Kelly et al., 2009, 2012). However, qualitative information about the experiences of people who resolve drug problems is much less available. Furthermore, much of what we know about recovery tends to be based on professional treatment perspectives. In other words, we know a lot about what professionals can effectively do about outcomes that *they* defined and much less about what those who recover need and *experience* (van der Stel, 2020).

Qualitative methods have proven to be valuable for such inquiries, as they can explore and understand human behavior from an emic approach (Whitley & Crawford, 2005). Qualitative studies on persons in drug treatment provided insights into both facilitating and contra-productive elements of treatment, through patients’ views about addiction service providers (Neale et al., 1998), barriers faced to access support (Copeland, 1997), and negative and positive experiences in or shortly after addiction treatment (Dekkers et al., 2019; Klingemann, 2011; Lock, 2004; Thom et al., 1992). Recently, studies focusing on recovery experiences outside of treatment settings have also started to emerge. One research group describes recovery as a developmental process from dependency and reactivity to personal autonomy and self-agency, in which continuing contact from services appear beneficial (Bjornestad et al., 2019; Svendsen et al., 2020). Klingeman (2012) found that developing better coping strategies for stress and craving contributed to sustained recovery from alcohol addiction. Mellor et al. (2020) uncovered a variety of socially shaped narratives about recovery, from persons who resolved an alcohol use problem without treatment or mutual aid support. Combining experiences from persons with and without addiction support, Dekkers et al. (2021) found that time and supportive environments facilitated recovery progress through developing a sense of self and future.

Still, literature on first-hand experiences—from persons who once had a drug addiction themselves—of long-term (multiple years after initiation of) drug addiction recovery, detached from any treatment setting or mutual aid support, is lacking. Consequently, our understanding of recovery processes detached from such settings is limited and varies depending on perspectives (e.g., professional, scientific, or lived experience). Since recovery is described as an individually unique long-term process, a better understanding of how processes of change occur in the full context of people’s lives may facilitate (evaluation or enhancement of) treatment, guide developments of new policies that support recovery in broad ways, and inform people with addiction and their family about what recovery pathways may look like over time. Therefore, to add to the current knowledge, this study aims to achieve a deeper understanding of how persons, in different stages of their recovery, experience drug addiction recovery in the Netherlands.

In the Netherlands, a range of inpatient, outpatient, and informal support services are available for people with addiction. Compared to other countries, the Netherlands can be characterized as having several progressive drug services that include drug consumption rooms, needle exchange, and heroin-assisted treatment. Addiction services may apply various philosophies around addiction and recovery and offer different types of interventions. Often, regional availability and professional networks determine which type of service a person gets referred to by his or her General Practitioner. There is no dominant model of addiction service. However, since 2010, the largest providers of addiction services in the Netherlands have embraced the emerging holistic concept of recovery (inspired by the mental health field) in their policies (Charter of Maastricht, 2010; Martinelli et al., 2022). The impact of this on recovery pathways is still unknown (Bellaert et al., 2021). The following research question guided our study: *How do people in different stages of recovery experience their recovery process from drug addiction over time?*

## Materials and Methods

### Design, Sample, and Recruitment

We recruited 30 persons from the Netherlands in recovery from illicit drug addiction—who self-identified as either “in recovery,” “had a drug addiction but not anymore,” or “have resolved a drug use problem”—and conducted a single in-depth interview with each of them. Although many aspects of both addiction and recovery are similar across licit and illicit substances, we focused on people with a history of illicit drug addiction because research on this subject is scarce compared to studies on alcohol addiction. Participants participated in two prior assessments from the REC-PATH cohort study (described in Best et al., 2018), aimed at mapping recovery pathways from the perspective of persons in recovery. Between January and June 2018, a convenience sample of 722 persons (of which 230 from the Netherlands) in recovery from drug addiction for 3 months or longer first completed the quantitative Life in Recovery survey (LiR) (Martinelli et al., 2020). We recruited them through social media, newsletters, conferences, recovery and addiction themed magazines, and printed flyers and posters. A subsample voluntarily left contact details so we could reach them for additional research. Demographics were collected through the LiR. For this study, we recruited a subsample aiming for an equal distribution in gender (15 men and 15 women) and self-attributed recovery stage (3 months to 1 year, *n* = 10; 1 to 5 years, *n* = 10; more than 5 years, *n* = 10) and strived for maximal variation in terms of age (mean of 38 years, range between 19 and 59 years old) and treatment history in order to cover a diverse sample. We stratified participants by key demographics, and then the first author randomly selected participants until a diverse sample was reached. We selected a heterogeneous sample in terms of treatment experiences, education level, and substances that were used problematically. The sample also contained persons with different levels of socio-economic status (e.g., some owned houses, while others resided in assisted living facilities or under debt restructuring). Furthermore, the sample contained two persons with a migration background (or non-Dutch ethnicity). Ethics approval was provided by the METC Erasmus MC in the Netherlands (MEC-2017-455).

Interviews took place in the summer of 2019 (1 year after initial recruitment) and lasted about 90 minutes (range: 80–110 minutes). The first author—an anthropologist and experienced qualitative researcher—conducted the interviews. He met with participants in their homes, at his office, or in a quiet bar or restaurant. Participants had spoken with him before when they participated in the cohort study and had given consent to participate and to be contacted further for the study. The first author and a research assistant approached 33 participants by telephone. Three participants did not respond, and none refused. We used the *lifeline interview* method, which allows for a retrospective lens to elicit autobiographical data covering personal recovery trajectories (Berends, 2011). Past work found that a timeline can be useful to advance the collection process and understanding of complex data (Berends, 2014; Monico et al., 2020; Sexton Topper & Bauermeister, 2021). We tested the interview on a person in recovery from alcohol addiction, so our full sample of persons in drug addiction recovery would remain available. The interviewe was a member of a client-representative organization advising the study. The test interview did not lead to significant changes in the protocol. Before each interview, the participant read and signed a consent form. During the interviews, the first author and participants (1) made a timeline of the participant’s life from the moment that their substance use “got out of control” until the present day on a paper sheet; (2) distinguished and chose periods on the timeline that were important for recovery to focus on in-depth; (3) discussed barriers and facilitators for recovery in those periods; and (4) discussed the meaning and definition of recovery. We included sample interview questions in Table 1. We conducted all interviews in Dutch. The interviews were audio recorded and transcribed verbatim. We entered 973 pages of transcripts into NVivo for coding. We translated quotes from the interviews from Dutch to English, and for readability purposes, we used pseudonyms throughout the results.

**Table 1.** Examples of Interview Questions.

Topic	Question/prompt
Opening questions: making a timeline	If you look back at the period between the starting point at which your drug use became problematic and where you stand today, what were meaningful positive or negative periods, moments or events?
	Which of those periods would you like to talk about first?
Looking at a specific period	Can you tell me more about that period?
	What things did you want to change during that period?
	Which things of that period did you want to keep? (Tangible or mindset)
Deepening questions	What helped you get ahead in that period?
	What was not at all helpful for you during that period?
	How was your social life during that period? Were you part of certain groups or communities?
	In what way was drug use a part of your daily life during that period (or not)?
End of interview	If you look at where you are now, what is important to you?
	If you look back at the timeline we discussed, how would you define your recovery?

### Data Analyses

Coding and analysis were guided by a seven-step inductive data-driven approach, based on Braun and Clarke’s (2006, 2012, 2023) guidelines for using thematic analysis. The first author familiarized himself with the data by reading the transcripts and his field notes several times while making rough notes. The field notes helped to recall the situation and setting of the interviews during analyses. He then reviewed the transcripts line-by-line and attached initial codes to the text in NVivo, which were refined and added to after discussions with the second author and by revisiting the interviews. For reflexivity purposes, the second and third authors each independently coded five transcripts. A group discussion provided many similar but also some alternative perspectives, which were used to develop themes. The first author looked for patterns and grouped the codes into themes. He created larger themes and sub-themes and discussed these during regular meetings with the second author. This helped to define and refine the themes and determine their relevance. The first author then wrote out the themes, using descriptive as well as interpretative elements. To ensure the structural validity of the findings and the inclusion of the most relevant themes (Hill et al., 1997), two more authors and one expert with first-hand recovery experience critically reviewed the findings and provided detailed feedback. Their comments helped to refine the themes further.

## Results

From the analyses, four main themes emerged that describe how participants experienced their recovery (presented in Table 1). Participants described processes in which they discovered that their addiction was connected to all other aspects of their life, not just their drug use (theme 1). While recovering, participants also started to reconsider their identity and look at their addiction experiences in a new light (theme 2). It further became clear that some processes of personal or social growth linked to recovery could span over several years (theme 3). Finally, participants discussed recovery experiences that can be very common or universal (theme 4)—experiences that anyone can have, regardless of drug addiction. The experiences of both men and women are reflected in each theme, and we found no major differences relating to the theme between men and women.

### Theme 1: Recovery Is a Broad Process of Change Because Addiction Is Interwoven With Everything

When talking about addiction and recovery, many participants described both states in terms like a *“mindset*,*” “attitude*,*”* or *“lifestyle*.*”* As such, a common experience among both men and women in our study is that recovery is a broad process of change beyond drug use because their addiction problems were also interwoven with their entire lives.

#### Recovery Is More Than Not Using Drugs

Some participants primarily saw drug use as their addiction problem. They expected to solve their problems by addressing this, which did not always work out.

What didn’t work out was to be much happier. (...) A bit happier, but not… The idea was that if I do this, it would be the end of… These are of course the dynamics of addiction. Oh, you take a drug, and you feel better all at once. It works the other way around too. Oh, I quit a drug in one go and then you will start feeling better again. But it doesn’t work that way at all. You are just the same asshole as when you were using. (Alexander, man, 59 years, >5 years recovery)

Alexander saw his expectation of “instant happiness” as part of his addiction. To learn and accept that there is no instant solution represented recovery to him. Similarly, Peter primarily worried about his drug use:

I wasn't sure what I wanted to change. I didn't want to use anymore because use always led to bullshit. So, I did what I had to do to avoid using. I didn't have a very clear idea of what I wanted differently, really, because I didn't know very well. (Peter, man, 45 years, >5 years recovery)

Peter said there were many times he wanted to stop using (e.g., each time he was released from prison). However, he kept failing until he broadened his attempts to change beyond his drug use and started to find other personal and social recovery goals. Most participants recognized this: “*The only thing I needed to change was everything*” (Ben, man, 47 years, 1–5 years recovery)*.*

Vice versa, other participants did not experience their drug use as problematic at first but experienced a range of other problems, including anxiety, stress, or burnout symptoms. Isabelle (woman, 53 years, >5 years recovery), for example, had stopped working for a while and was using a combination of illicit and prescription drugs while lying in bed all day. She only realized that there was a link with her drug use after visiting a psychiatrist for other symptoms. In retrospect, she found it odd that it took her so long to see this link. She, and other participants in similar situations, thought this delayed realization was due to negative stereotypes of *“drug addicts*.*”* They assumed that if you manage to sustain a home, a family, or a job, there is *“no way”* that you can be addicted.

#### Things to Recover: Examples of Broader Recovery Goals

Daisy (woman, 30 years, 1–5 years recovery) further emphasized: *“You can be clean, and you can be in recovery. But being clean doesn’t work for me. I tried that*.*”* While in a rehab center, she was able to not use quite successfully for a while, by avoiding triggers that induced cravings. However, eventually, she was sent out for dating another patient. A relapse followed and she felt worse than before. This experience made her realize that the way she engaged in relationships with men was problematic, as she put herself and her recovery in second place. Peter (man, 45 years, >5 years recovery) also mentioned his relationships with women as part of his addiction behavior that continued long into his recovery from drug use. He joined a Sex and Love Addiction Anonymous 12-step group to work on this to further his recovery.

Isabelle eventually focused on her drug use and managed to stop successfully. However, her situation was not improving:

I got into serious dislike with my employer. That ended up with me being fired. At one point I was like ‘okay, I'm clean’, that first exercise was over. But I'm starting to feel worse and worse. (Isabelle, woman, 53 years, >5 years recovery)

She continued to search for help and was eventually diagnosed with autism. She learned that she was self-medicating to dampen the excessive stimuli she experienced due to autism. This helped her to develop other strategies to dampen stimuli, which reduced her craving for drugs and helped her to gradually feel better.

We encountered participants with a broad range of recovery goals. These ranged from practical goals, such as *“day routines,” “financial stability,”* and *“acquiring a job,”* to emotional goals, including *“becoming happier”* and acquiring *“a sense of peace”* or *“serenity.”* Such goals appeared particularly important in the initial stage of recovery. Yvette, in the first year of her recovery, needed practical goals *“because, why would you get up if you don’t have a goal, anyway?”* (Yvette, woman, 26 years, <1 year recovery). Manuel, on the other hand, had been homeless for 11 years and was not looking for something to do but aimed to achieve serenity instead. He said:

Yeah, I really wanted rest man. Because I was always running, everywhere. There was so much unrest. (Man, 47 years, 1–5 years recovery)

He described this as a paradoxical feeling: *“to do nothing to improve your situation*.*”*

For many participants, achieving goals equaled *“doing good”* or *“living well,”* which resulted in feeling more *“real”* or *“authentic.”* Daisy, for example, noted that she does not *“feel fake anymore”* (woman, 30 years, 1–5 years recovery). Participants further linked achieving broad goals with *“being part of society”* and *“doing your part”*:

That now, I am on the train and I’m going to work with all the other working people. So that now I *am* becoming a productive member of society. Yes, look at me! (Kyle, man, 42 years, 1–5 years recovery)

### Theme 2: Recovery Is Reconsidering Identity, Seeing Things in a New Light

At the end of each interview, we asked participants to describe what recovery means to them. Many participants, both men and women, would then describe how they saw themselves and their (current and past) behavior in a new light after initiating recovery. For some, this was about *“listening to myself,” “considering my needs,”* and *“staying close to myself.”* For others, it concerned learning about and accepting certain vulnerabilities, or adopting a non-user identity.

For Manuel (man, 47 years, 1–5 years recovery), drugs had led his life and would take over his life again if he ever started using again. It was important to close that door permanently in his mind and to change his identity from a drug user to a non-user. This was a recurring theme for other participants as well. Some had relapsed after trying substances again after some time of abstinence or replaced one substance with another. They considered themselves vulnerable to any substance use. Accepting this vulnerability was felt as a *“liberation”* or an improvement by some. Yara, instead, felt it as a *“grieving”* process:

In the beginning, I thought, well I'm going to do this for a while because, yeah, how can you never celebrate your birthday again without...? Look, I get that the drugs need to be gone, but New Year's Eve and everything without anything? How? (Yara, woman, 36 years, <1 year recovery)

She felt like this mostly at the beginning of her recovery but had now accepted it better. Adopting this new identity was also relational, as she was now viewed by and had to explain herself to others as a *non-user* of alcohol and drugs. Her experience illustrates that while drug and alcohol use has meaning to the user, it is also a social and contextually sensitive practice.

Other participants also reconsidered past behavior and events. Edwin, for example, saw who he was during his addiction experience in a new light after being diagnosed by a professional.

I always thought I’m weird, I’m not right, I’m crazy. That’s why you get aggressive, you go against everything. Then I was like, it’s normal. This behavior is normal. (...) It’s in my brain, not in my character. (Edwin, man, 48 years, >5 years recovery)

He always knew that his drug use was a way to alleviate his busy mind. But now he learned that his busy mind came from an attention deficit hyperactivity disorder. He further learned that childhood traumas affected his behavior later in life. Knowing that this behavior came from something that happened *to* him, and not from some character flaw, helped him to look at his addiction experiences with more compassion. This helped him to adopt a more positive recovery identity.

### Theme 3: Recovery Is a Staged Long-Term Process

We collected multiple experiences from participants that indicate that certain addiction recovery processes can span over several years. We further asked participants to distinguish different periods in their life and to characterize them. This allowed participants to reflect on their recovery from a long-term perspective and describe how past events shaped later experiences.

#### Planting a Seed

For many participants, there could be years between the moment at which they *wanted* to change their situation and before they were able to do so. Still, some described this early awareness of wanting to change as a *planted seed* for recovery. Seeds often came from life-impacting events, such as the birth of a child, receiving treatment, becoming homeless, being in or ending an unhealthy relationship, meeting persons with similar lived experiences, or a judicial punishment or measure.

Daisy explained how she relapsed a few months after treatment when she was unable to bear the precarious situation of her child’s hospitalization. However, her *“failed attempt”* at recovery was not a waste and eventually helped her initiate recovery again:

I had just tasted enough of recovery, or at least the feeling I had when I was sober. (Daisy, woman, 30 years, 1–5 years recovery)

To Daisy, the process of recovery had germinated after that experience and could sprout from that later.

#### Stages of Recovery

Participants, particularly those with multiple years of recovery experience, also distinguished stages in their recovery. This became apparent when we asked participants to distinguish periods on the lifeline interview sheet, but also when they reflected on what their recovery meant to them *“now.”* While the length and order of some stages could vary, some similarities became clear in the analysis. Many participants described beginning their recovery with a period to “*stabilize,”* for example:

Especially in the beginning, you have the idea that you are standing there with a big spotlight on you, that completely dazzles you. And it takes quite a long time before you get used to that. (...) And at a certain point, you get the overview again, but that is a whole process that you go through. (Jolien, woman, 30 years, 1–5 years recovery)

Reflecting on this first stage, participants often explained how difficult it was to maintain other aspects of life. In this context, some participants who were 12-step group members cited the “*90 meetings in 90 days*” principle: the program’s advice to join at least one meeting a day in the first 90 days. Residential treatment facilities, where participants were disengaged from daily worries and tasks to solely focus on treatment, also had this early intensity.

On the other hand, Sara argued that she needed time and space in the beginning, not an intense regime. As a metaphor, she compared her recovery to taking care of a wound:

You also must make sure that your wound can breathe. That comes first. Because if you just put a plaster on it, it won't do the trick either. (...) Giving someone the space to take care of it and let them know: ‘look, this hurts’. (Sara, woman, 54 years, >5 years recovery)

Ben added that it had helped him to not work in the first stage of his recovery:

If you don’t have to work, don’t do it, and really focus on your recovery, that’s already a full-time job. A roof over your head, food… the rest will all come later. (Ben, man, 47 years, 1–5 years recovery)

Either needing intensity or time and space indicates a certain fragility in early recovery. At the same time, some participants paradoxically described the early stage of recovery as *“sitting on a pink cloud,”* referring to powerful positive energy and feeling good. This feeling was the result of taking control over elements in life that seemed uncontrollable before. Still, the pink cloud also came with risks:

Then I would also reconcile with four to five people in one week. Almost every day going to someone to do penance. Then they said to me: “Yes, that can be toned down a bit. Why don’t you divide it over five years, isn't that okay too?” (Simon, man, 35 years, <1 year recovery)

Simon became too zealous in his recovery and overdid things, exhausting himself and others around him. Another difficult moment was when the newly found energy gradually became “*normal*”:

I felt a lot of love in myself and around me. I heard the birds whistle. (...) But when I look at the last year, it has become a bit more normal. When I am occasionally with people around me, who hear my story for the first time or I talk about it, I notice that it no longer affects me in the same way as before. (Simon, man, 35 years, <1 year recovery)

Simon felt like he was not progressing as fast as before. Several participants describe that after *“the pink cloud”* they had to find other sources of enthusiasm and energy to further recovery.

Experiences of later recovery stages are more diffuse and unique for participants:

As my recovery progressed, I also began to address other facets of recovery. (..) Going back to look for work. Becoming financially stable. (Willem, man, 28 years, <1 year recovery)

Depending on their situation, participants started aiming their attention, energy, and time toward other aspects of life, such as work, study, or a romantic relationship. While such things were described as *“distractions”* in the early recovery stage—sometimes even linked to relapse—they characterize subsequent stages of recovery.

#### Recovering or Recovered?

Participants with more than 5 years of recovery experience had different views about whether they were still “in recovery” or “recovered.” Paul (man, 41 years, >5 years recovery) said that he was still in recovery and kept *“peeling away layers of the onion*.*”* While he had already dealt with many things, he still found issues that he related to his recovery to work on. Other participants felt they had to *“move on.”* Kees, for example, worked in prevention. He described how, lately, he wanted to distance himself more from his past:

I don’t feel like talking about the Kees I was then. I have benefited a lot from that for a while, also you know, providing information for others, but it bothers me a lot now. (Kees, man, 38 years, 1–5 years recovery)

Others also noted that, after following a certain treatment regime or mutual aid program, they felt the need to *“break free*” from that after some time. Some participants developed their *“own method”* for recovery or became *“rebellious”* or *“stubborn.”* Sara, with more than 5 years of recovery experience, noted how she sometimes saw other people struggle when they *“stick too much”* with what they were taught:

Then I think to myself ‘just let it go, man’. If you're in your recovery and things are going well, let go of those steps at some point. But people are so afraid of relapsing. (woman, 54 years, >5 years recovery)

### Theme 4: Universal Life Processes Are Part of Recovery

Besides addiction and recovery, participants dealt with a variety of life events. This often makes causes and effects linked to these experiences non-linear. In this context, participants described recovery processes that can be interpreted as *universal* or *“normal”* developmental processes. Processes that many people may experience, regardless of past addiction.

#### Coming of Age

As such, some participants described a process similar to the normative idea of entering adulthood or coming of age.

I can now genuinely enjoy sitting on the couch on a Saturday night and putting on a movie. I am now mainly concerned with what I like, just… It’s very different. Social life is… I still have it, but it’s just in a different way. (Angelina, woman, 36 years, <1 year recovery)

When we asked Angelina why she described her social life as different, she reflected on her life course and age: *“I’m 36 now*.*”* It became more important for her to listen to herself, instead of relating to peers, representing a shifting self-perception. Jolien further illustrates this by sharing how she implemented boundaries to not overstimulate herself as part of her recovery:

I used to just put 10 appointments in my calendar, you know? I went everywhere, I was doing everything. And (...) then I came right back home, and I was completely over-stimulated and stuff. I don't do any of that anymore. (Woman, 30 years, 1–5 years recovery)

We also found changes in self-perception related to drug use behavior that came with aging. Giovanni, in recovery from cannabis addiction, told us that he had recently smoked cannabis again. However, this was not like his addicted use:

I have learned so much as a person, not just in recovery. (...) Your perception changes, hasn’t yours too? Don't you think differently about things than when you were 18? I notice that in myself too. I used to dive into everything and now I'm like ‘shit, if I do this, then this could happen’. You just change. (Giovanni, man, 35 years, 1–5 years recovery)

Giovanni thinks this happens to everyone who gets older. He *“just happened”* to experience some things on this path that most people will not, but the general process of change is similar.

#### This Should Be Taught in Schools

Additionally, participants applied specific recovery experiences broadly in their lives, beyond the context of addiction. They described, for example, how other people without addiction experiences could benefit from their recovery experiences. Angelina (woman, 36 years, <1 year recovery) said that she sometimes shares her recovery experiences with her parents, such as being self-reflective, open, and honest about inner experiences. Her parents also *“get something out of it.”* Furthermore, participants in mutual aid recovery groups regarded the experience where peers *“really listen”* to each other *“free of judgment”* and *“share”* as something that anyone could benefit from:

Why are there not such groups for people who feel alone or who are depressed, or you name it? (...) Because it is also about struggles or pitfalls or things you run into. Normal people, non-addicts, have this too. (Simon, man, 38 years, <1 year recovery)

Ben (man, 47 years, 1–5 years recovery) underlined this. He said that the principles of mutual aid groups, such as self-reflection, fulfill such a universal need that they *“should be taught at elementary schools.”* The social connection and willingness to help others is something the world needs more of, according to Ben. He added that besides the healing potential of such experiences, they may also work preventive, boosting resilience for many potentially difficult life events.

## Discussion

The goal of this study was to achieve a deeper understanding of drug addiction recovery experiences over time. Although these experiences were multifaceted and heterogenous among participants, we found four central themes: (1) addiction is connected to all aspects of participants’ lives beyond just drug use; (2) participants reconsidered their identity and looked at their addiction experiences in a new light; (3) recovery processes can span several years; and (4) common or universal experiences can be connected to addiction recovery.

### Interwoven Recovery

Overall, the four themes support that addiction relates to various aspects of the individual and social environment, and thus, that recovery can take place in many life domains (Laudet & White, 2010; Mellor et al., 2020; Moos & Moos, 2007). From the autobiographic data, we could distill how recovery was interwoven with the entire lives of participants (Bellaert, 2022). In addition, we show that some (universal) recovery processes transcend the context of addiction. Research on stigma shows that focusing on deficits, while neglecting resilience, capacity, and humanity, reinforces the devaluation of people with drug addiction (del Vecchio, 2006). Thus, recognizing the commonness of some recovery experiences, without understating the impact of drug addiction, may help to reduce stigmatization and othering. This in turn may facilitate recovery, as stigmatization and subsequent discrimination are considered major barriers to treatment and recovery (Davidson et al., 2006; Goodyear et al., 2021; van Weeghel et al., 2019). More research on the broad experiences of people in addiction recovery is therefore needed. Results of this can be used in policy efforts disseminating first-hand recovery experiences as helpful and hopeful sources of information.

### Identity Change

In line with other research, we also found that participants in recovery had changed how they looked at themselves and their past experiences (Dahl, 2015; Dekkers et al., 2019; Hill & Leeming, 2014; Liebregts et al., 2015; Mackintosh & Knight, 2012). This self-narrative development helped participants to understand themselves better and construct new identities (McIntosh & McKeganey, 2000). Furthermore, identity change was also social (Bathish et al., 2017; Best et al., 2016; Dingle et al., 2019), as participants reconsidered their identity in relation to others and to their place in society. Identity is also one of the key processes in the CHIME (Connectedness, Hope, Identity, Meaning, and Empowerment) framework, built on a systematic review of mental health recovery literature (Leamy et al., 2011). This can be placed within the wider concept of *personal recovery* which originated in the mental health field but is also applied to addiction recovery (Anthony, 1993; Davidson & White, 2007; de Ruysscher et al., 2017). Personal recovery is understood to drive recovery on clinical, functional, and societal outcomes and includes giving meaning to past events, (re)gaining control over one’s own life, and forming a new identity to (re)establish personal and social values (van der Stel, 2013). Similarly, participants in our study describe that recovery meant seeing themselves in a new light.

### A Long-Term Evolving Process

We found no specific order in which recovery experiences occurred, but our results suggest that recovery is a staged long-term process. We found, for example, how participants link past life events to positive changes later in life, described as *planting a seed* of recovery. Similarly, research describes a cumulative effect of multiple treatment and support experiences that can develop over years (Dennis et al., 2007; Hser et al., 1997) and that contextual dynamics—rather than single events—determine whether certain life events facilitate change (Bellaert et al., 2022; Patton et al., 2022). Our findings also show that participantsexperienced stages in their recovery, for example, a period to *“stabilize”* at the beginning of their recovery, in which it helped to focus on recovery and to reduce distractions such as work or romantic relations. This is in line with research showing that processes of identity change consolidate over time and that, for example, engagement with the AA-groups and associated behaviors are not as needed to maintain a recovery identity after some time (Frings et al., 2021). An explanation may be that when persons become accustomed (or trained) to recovery and related behavior, this then becomes more automatic and easier to maintain (Lindgren et al., 2017; Wiers et al., 2010). At the same time, participants described needing to find other sources of enthusiasm and energy after their recovery became more normal. This may suggest that participants did not trust this automatic state completely. It also indicates that support needs change over time, as participants started to focus more on broader and more diffuse aspects of their lives. Combined, this means that addiction treatment and support should orient toward long-term goals, instead of the currently dominant acute model of care (DuPont et al., 2015; Martinelli et al., 2020; Scott et al., 2021).

### Common Processes

The common processes, such as coming of age, are also described in the addiction literature. A popular explanation of why people stop or reduce their drug use is the idea of *maturing out*, for example. Winick (1962) noted how persons who became addicted to heroin in their late teens eventually stopped using as they got older and took on *adult roles*, which had been avoided before. Others criticized this notion for being too simplistic, vague, and imprecise because Winick’s theory assumed that drug use and addiction are immature behaviors (Waldorf & Biernacki, 1981). As a sole explanation of why people initiate recovery, maturing out is indeed too simplistic. However, participants’ recovery experiences resemble much of the normative ideas about maturing or becoming an adult, such as appreciating rest over activities, reducing risk behaviors, and learning about your own preferences.

### Strengths and Limitations

A strength of this study is that we purposively selected participants in varying recovery stages, of varying ages, and an equal number of men and women. Since we did not recruit from a specific treatment setting, the current sample covers a broad arrange of recovery experiences that supersede that of studies that recruited from one setting. We recruited from an ongoing cohort study with a convenience sample, which may have led to a degree of selection bias. By only including persons who are willing and able to take part in a cohort study about addiction recovery, we may have excluded persons with (mild) cognitive or intellectual disabilities or persons in vulnerable situations that do not allow for participation, such as imprisonment. Data-driven thematic analysis involves higher level conceptual interpretation, inherent to the coding process, which may be seen as a limitation because other researchers may allocate different code structures and deduct different interpretations of the same data. By reflecting on and discussing the analyses regularly with the coauthors, we strengthened the validity of these interpretations. Furthermore, using the timeline with the in-depth interview helped to concisely represent complex autobiographical data, including the perceptions and experiences of participants. By drawing out periods and noting down events during the interview, the researcher and participant were able to check whether data were recorded correctly and to discuss them in the context of the participant’s life, sometimes referring back and forth between periods and events that were connected. While research suggests that there are differences in the recovery experience of women and men (Andersson et al., 2021; van Steenberghe et al., 2021), we did not find any notable differences in the four main themes. On the one hand, this may mean that we were able to capture experiences that supersede intersections with gender or that there are only limited differences between genders in our sample. On the other hand, it could mean that we were not attentive enough to gender differences. Future research focused specifically on comparing recovery experiences across gender identities, including minority groups (LGBTQIA+), is therefore necessary. Similarly, the impact of ethnicity on recovery experiences should be explored further (Goldbach et al., 2014). Lastly, our findings primarily involve individual experiences of recovery, relating to identity and self-fulfillment. Participants also discussed basic physiological and safety needs (Maslow, 1943) and structural and social factors that influenced their recovery pathways, such as homelessness, detention, stigma, or welfare opportunities, but to a lesser extent. This may mean that basic needs were already fulfilled or that recovery transcends basic needs. It may also have been a result of using the lifeline interview that focuses on autobiographical (individual) information, or the result of internalized societal notions that highlight individual experiences and responsibility regarding addiction in society and treatment settings (Fomiatti et al., 2017; Lancaster et al., 2015). Lastly, Bellaert (2022) argues that recovery is also a contextualized process that is influenced by the social environment and broader society. Besides focusing on what individuals do in recovery, it is therefore also important to consider how environments can support recovery.

### Implications for Clinical Practice

• Addiction treatment and support should orient toward long-term tailored goals, instead of the currently dominant acute model of care.
• Recognizing the commonness of some recovery experiences without understating the impact of drug addiction may help to reduce stigmatization and othering, which in turn may facilitate recovery.
• Disseminating first-hand recovery experiences may function as helpful and hopeful sources of information for persons in early recovery, those seeking to initiate recovery, or their families, partners, and friends.

### Conclusions

Our study contributes to the expanding recovery literature by providing insights into how people with drug addiction experience recovery over time. Because our sample is highly heterogenous and recruited from a variety of settings, our findings provide recovery experiences that supersede those of studies performed in a particular treatment setting. We found that recovery involves an interwoven long-term process, including identity change and common or universal life processes. This means that policies and clinical practice should be aimed at supporting long-term tailored recovery goals. Disseminating first-hand recovery experiences may also help to reduce stigmatization and othering and function as helpful and hopeful sources of information for persons in early recovery, those seeking to initiate recovery, or their families, partners, and friends.

## Supplemental Material

Supplemental Material - Understanding the Process of Drug Addiction Recovery Through First-Hand Experiences: A Qualitative Study in the Netherlands Using Lifeline Interviews Click here for additional data file. Supplemental Material for Understanding the Process of Drug Addiction Recovery Through First-Hand Experiences: A Qualitative Study in the Netherlands Using Lifeline Interviews by T. F. Martinelli, D. P. K. Roeg, L. Bellaert, D. van de Mheen, and G. E. Nagelhout in Qualitative Health Research.

## References

[bibr1-10497323231174161] Andersson C. Wincup E. Best D. Irving J. (2021). Gender and recovery pathways in the UK. Drugs: Education, Prevention and Policy, 28(5), 454–464. 10.1080/09687637.2020.1852180

[bibr2-10497323231174161] Anthony W. A. (1993). Recovery from mental illness: The guiding vision of the mental health service system in the 1990s. Psychosocial Rehabilitation Journal, 16(4), 11–23. 10.1037/h0095655

[bibr3-10497323231174161] Bathish R. Best D. Savic M. Beckwith M. Mackenzie J. Lubman D. I. (2017). Is it me or should my friends take the credit?” The role of social networks and social identity in recovery from addiction. Journal of Applied Social Psychology, 47(1), 35–46. 10.1111/jasp.12420

[bibr4-10497323231174161] Bellaert L. (2022). Addiction recovery: Towards a contextualized understanding. Gompel and Svacina.

[bibr5-10497323231174161] Bellaert L. Martinelli T. F. Vanderplasschen W. Best D. van de Mheen D. vander Laenen F. (2021) Chasing a pot of gold: An analysis of emerging recovery-oriented addiction policies in flanders (Belgium) and The Netherlands. Drugs: Education, Prevention and Policy, 28(5), 399–410. 10.1080/09687637.2021.1915250

[bibr6-10497323231174161] Bellaert L. van Steenberghe T. de Maeyer J. vander Laenen F. Vanderplasschen W. (2022). Turning points toward drug addiction recovery: Contextualizing underlying dynamics of change. Addiction Research and Theory, 30(4), 294–303. 10.1080/16066359.2022.2026934

[bibr7-10497323231174161] Berends L. (2011). Embracing the visual: Using timelines with in-depth interviews on substance use and treatment. Qualitative Report, 16(1), 1–9. 10.46743/2160-3715/2011.1036

[bibr8-10497323231174161] Berends L. (2014). Embracing the visual: Using timelines with in-depth interviews on substance use and treatment. The Qualitative Report, 16(1), 1–9. 10.46743/2160-3715/2011.1036

[bibr9-10497323231174161] Best D. Beckwith M. Haslam C. Alexander Haslam S. Jetten J. Mawson E. Lubman D. I. (2016). Overcoming alcohol and other drug addiction as a process of social identity transition: The social identity model of recovery (SIMOR). Addiction Research and Theory, 24(2), 111–123. 10.3109/16066359.2015.1075980

[bibr10-10497323231174161] Best D. Gow J. Knox T. Taylor A. Groshkova T. White W. (2012). Mapping the recovery stories of drinkers and drug users in Glasgow: Quality of life and its associations with measures of recovery capital. Drug and Alcohol Review, 31(3), 334–341. 10.1111/j.1465-3362.2011.00321.x 21615809

[bibr11-10497323231174161] Best D. Hennessy E. A. (2022). The science of recovery capital: Where do we go from here? Addiction, 117(4), 1139–1145. 10.1111/add.15732 34729852 PMC9209877

[bibr12-10497323231174161] Best D. Savic M. Bathish R. Edwards M. Irving J. Cano I. Albertson K. (2018). Life in recovery in Australia and the United Kingdom: Do stages of recovery differ across national boundaries? Alcoholism Treatment Quarterly, 36(4), 530–541. 10.1080/07347324.2018.1492336

[bibr13-10497323231174161] Best D. Vanderplasschen W. Nisic M. (2020). Measuring capital in active addiction and recovery: The development of the strengths and barriers recovery scale (SABRS). Substance Abuse Treatment, Prevention, and Policy, 15(1), 40–48. 10.1186/s13011-020-00281-7 32546171 PMC7298842

[bibr14-10497323231174161] Best D. Vanderplasschen W. Van de Mheen D. De Maeyer J. Colman C. Vander Laenen F. Irving J. Andersson C. Edwards M. Bellaert L. Martinelli T. Graham S. Hamer R. Nagelhout G. E. (2018). REC-PATH (recovery pathways): Overview of a four-country study of pathways to recovery from problematic drug use. Alcoholism Treatment Quarterly, 36(4), 517–529. 10.1080/07347324.2018.1488550

[bibr88-10497323231174161] Bjornestad . 10.3389/fpsyt.2019.00689

[bibr15-10497323231174161] Braun V. Clarke V. (2006). Using thematic analysis in psychology. Qualitative Research in Psychology, 3(2), 77–101. 10.1191/1478088706qp063oa

[bibr16-10497323231174161] Braun V. Clarke V. (2012). Thematic analysis. In APA handbook of research methods in psychology. Research designs: Quantitative, qualitative, neuropsychological, and biological (Vol 2, pp. 57–71). American Psychological Association. 10.1037/13620-004

[bibr17-10497323231174161] Braun V. Clarke V. (2023, February 3). Thematic analysis. The University of Auckland. https://www.Thematicanalysis.Net/

[bibr18-10497323231174161] Charter of Maastricht , (2010). Black Hole Foundation (2010)

[bibr19-10497323231174161] Cloud W. Granfield R. (2008). Conceptualizing recovery capital: Expansion of a theoretical construct. Substance Use and Misuse, 43(12–13), 1971–1986. 10.1080/10826080802289762 19016174

[bibr20-10497323231174161] Copeland J. (1997). A qualitative study of barriers to formal treatment among women who self-managed change in addictive behaviours. Journal of Substance Abuse Treatment, 14(2), 183–190. 10.1016/S0740-5472(96)00108-0 9258863

[bibr21-10497323231174161] Dahl S. L. (2015). Remaining a user while cutting down: The relationship between cannabis use and identity. Drugs: Education, Prevention and Policy, 22(3), 175–184. 10.3109/09687637.2014.920765

[bibr22-10497323231174161] Davidson L. O’Connell M. Tondora J. Styron T. Kangas K. (2006). The top ten concerns about recovery encountered in mental health system transformation. Psychiatric Services, 57(5), 640–645. 10.1176/ps.2006.57.5.640 16675756

[bibr23-10497323231174161] Davidson L. White W. (2007). The concept of recovery as an organizing principle for integrating mental health and addiction services. The Journal of Behavioral Health Services and Research, 34(2), 109–120. 10.1007/s11414-007-9053-7 17351758

[bibr24-10497323231174161] Deegan P. E. (1988). Recovery: The lived experience of rehabilitation. Psychosocial Rehabilitation Journal, 11(4), 11–19. 10.1037/h0099565

[bibr25-10497323231174161] Dekkers A. Bellaert L. Meulewaeter F. de Ruysscher C. Vanderplasschen W. (2021). Exploring essential components of addiction recovery: A qualitative study across assisted and unassisted recovery pathways. Drugs: Education, Prevention and Policy, 28(5), 486–495. 10.1080/09687637.2021.1943315

[bibr26-10497323231174161] Dekkers A. de Ruysscher C. Vanderplasschen W. (2019). Perspectives of cocaine users on addiction recovery: A qualitative study following a CRA + vouchers programme. Drugs: Education, Prevention and Policy, 27(4), 282–296. 10.1080/09687637.2019.1687647

[bibr27-10497323231174161] del Vecchio P. (2006). Commentary: All we are saying is give people with mental illnesses a chance. Psychiatric Services, 57(5), 646–646. 10.1176/ps.2006.57.5.646 16675757

[bibr28-10497323231174161] Dennis M. L. Foss M. A. Scott C. K. (2007). An eight-year perspective on the relationship between the duration of abstinence and other aspects of recovery. Evaluation Review, 31(6), 585–612. 10.1177/0193841X07307771 17986709

[bibr29-10497323231174161] Dennis M. L. Scott C. K. Laudet A. (2014). Beyond bricks and mortar: Recent research on substance use disorder recovery management. Current Psychiatry Reports, 16(4), 442. 10.1007/s11920-014-0442-3 24557873 PMC5715665

[bibr30-10497323231174161] de Ruysscher C. Vandevelde S. Vanderplasschen W. de Maeyer J. Vanheule S. (2017). The concept of recovery as experienced by persons with dual diagnosis: A systematic review of qualitative research from a first-person perspective. Journal of Dual Diagnosis, 13(4), 264–279. 10.1080/15504263.2017.1349977 28699834

[bibr31-10497323231174161] Dingle G. A. Haslam C. Best D. Chan G. Staiger P. K. Savic M. Beckwith M. Mackenzie J. Bathish R. Lubman D. I. (2019). Social identity differentiation predicts commitment to sobriety and wellbeing in residents of therapeutic communities. Social Science and Medicine, 237(277–9536), 112459. 10.1016/j.socscimed.2019.112459 31404883

[bibr32-10497323231174161] DuPont R. L. Compton W. M. McLellan A. T. (2015). Five-year recovery: A new standard for assessing effectiveness of substance use disorder treatment. Journal of Substance Abuse Treatment, 58, 1–5. 10.1016/j.jsat.2015.06.024 26277423

[bibr33-10497323231174161] Fomiatti R. Moore D. Fraser S. (2017). Interpellating recovery: The politics of ‘identity’ in recovery-focused treatment. The International Journal on Drug Policy, 44(955–3959), 174–182. 10.1016/j.drugpo.2017.04.001 28511843

[bibr34-10497323231174161] Frings D. Wood K. v. Albery I. P. (2021). New converts and seasoned campaigners: The role of social identity at different stages in the addiction recovery journey. Drugs: Education, Prevention and Policy, 28(5), 496–503. 10.1080/09687637.2021.1914551

[bibr35-10497323231174161] Goldbach J. T. Tanner-Smith E. E. Bagwell M. Dunlap S. (2014). Minority stress and substance use in sexual minority adolescents: A meta-analysis. Prevention Science: The Official Journal of the Society for Prevention Research, 15(3), 350–363. 10.1007/s11121-013-0393-7 23605479

[bibr36-10497323231174161] Goodyear T. Brown H. Browne A. J. Hoong P. Ti L. Knight R. (2021). Stigma is where the harm comes from”: Exploring expectations and lived experiences of hepatitis C virus post-treatment trajectories among people who inject drugs. The International Journal on Drug Policy, 96(955–3959), 103238. 10.1016/j.drugpo.2021.103238 33902968 PMC8881088

[bibr37-10497323231174161] Groshkova T. Best D. White W. (2013). The Assessment of Recovery Capital: Properties and psychometrics of a measure of addiction recovery strengths. Drug and Alcohol Review, 32(2), 187–194. 10.1111/j.1465-3362.2012.00489.x 22882622

[bibr38-10497323231174161] Helm P. (2019). Sobriety versus abstinence. How 12-stepper negotiate long-term recovery across groups. Addiction Research and Theory, 27(1), 29–36. 10.1080/16066359.2018.1530348

[bibr39-10497323231174161] Hennessy E. A. (2017). Recovery capital: A systematic review of the literature. Addiction Research and Theory, 25(5), 349–360. 10.1080/16066359.2017.1297990

[bibr40-10497323231174161] Hibbert L. J. Best D. W. (2011). Assessing recovery and functioning in former problem drinkers at different stages of their recovery journeys. Drug and Alcohol Review, 30(1), 12–20. 10.1111/j.1465-3362.2010.00190.x 21219492

[bibr41-10497323231174161] Hill C. E. Thompson B. J. Williams E. N. (1997). A guide to conducting consensual qualitative research. The Counseling Psychologist, 25(4), 517–572. 10.1177/0011000097254001

[bibr42-10497323231174161] Hill J. v. Leeming D. (2014). Reconstructing ‘the alcoholic’: Recovering from alcohol addiction and the stigma this entails. International Journal of Mental Health and Addiction, 12(6), 759–771. 10.1007/s11469-014-9508-z

[bibr43-10497323231174161] Hser Y.-I. Anglin M. D. Grella C. Longshore D. Prendergast M. L. (1997). Drug treatment careers A conceptual framework and existing research findings. Journal of Substance Abuse Treatment, 14(6), 543–558. 10.1016/S0740-5472(97)00016-0 9437626

[bibr44-10497323231174161] Hser Y.-I. Longshore D. Anglin M. D. (2007). The life course perspective on drug use: A conceptual framework for understanding drug use trajectories. Evaluation Review, 31(6), 515–547. 10.1177/0193841X07307316 17986706

[bibr45-10497323231174161] Kaskutas L. A. Borkman T. J. Laudet A. Ritter L. A. Witbrodt J. Subbaraman M. S. Stunz A. Bond J. (2014). Elements that define recovery: The experiential perspective. Journal of Studies on Alcohol and Drugs, 75(6), 999–1010. 10.15288/jsad.2014.75.999 25343658 PMC4211341

[bibr46-10497323231174161] Kelly J. F. Bergman B. Hoeppner B. B. Vilsaint C. White W. L. (2017). Prevalence and pathways of recovery from drug and alcohol problems in the United States population: Implications for practice, research, and policy. Drug and Alcohol Dependence, 181(376–8716), 162–169. 10.1016/j.drugalcdep.2017.09.028 29055821 PMC6076174

[bibr47-10497323231174161] Kelly J. F. Hoeppner B. (2015). A biaxial formulation of the recovery construct. Addiction Research and Theory, 23(1), 5–9. 10.3109/16066359.2014.930132

[bibr48-10497323231174161] Kelly J. F. Hoeppner B. Stout R. L. Pagano M. (2012). Determining the relative importance of the mechanisms of behavior change within alcoholics anonymous: A multiple mediator analysis. Addiction, 107(2), 289–299. 10.1111/j.1360-0443.2011.03593.x 21917054 PMC3242865

[bibr49-10497323231174161] Kelly J. F. Magill M. Stout R. L. (2009). How do people recover from alcohol dependence? A systematic review of the research on mechanisms of behavior change in alcoholics anonymous. Addiction Research and Theory, 17(3), 236–259. 10.1080/16066350902770458

[bibr50-10497323231174161] Klingemann J. I. (2011). Lay and professional concepts of alcohol dependence in the process of recovery from addiction among treated and non-treated individuals in Poland: A qualitative study. Addiction Research and Theory, 19(3), 266–275. 10.3109/16066359.2010.520771

[bibr51-10497323231174161] Klingemann J. I. (2012). Mapping the maintenance stage of recovery: A qualitative study among treated and non-treated former alcohol dependents in Poland. Alcohol and Alcoholism, 47(3), 296–303. 10.1093/alcalc/agr163 22271909

[bibr52-10497323231174161] Lancaster K. Duke K. Ritter A. (2015). Producing the ‘problem of drugs’: A cross national-comparison of ‘recovery’ discourse in two Australian and British reports. The International Journal on Drug Policy, 26(7), 617–625. 10.1016/j.drugpo.2015.04.006 25962733

[bibr53-10497323231174161] Laudet A. B. (2007). What does recovery mean to you? Lessons from the recovery experience for research and practice. Journal of Substance Abuse Treatment, 33(3), 243–256. 10.1016/j.jsat.2007.04.014 17889296 PMC2083562

[bibr54-10497323231174161] Laudet A. B. (2008). The road to recovery: Where are we going and how do we get there? Empirically driven conclusions and future directions for service development and research. Substance Use and Misuse, 43(12–13), 2001–2020. 10.1080/10826080802293459 19016176 PMC2593852

[bibr55-10497323231174161] Laudet A. B. White W. (2010). What are your priorities right now? Identifying service needs across recovery stages to inform service development. Journal of Substance Abuse Treatment, 38(1), 51–59. 10.1016/j.jsat.2009.06.003 19631490 PMC2789901

[bibr56-10497323231174161] Laudet A. B. White W. L. (2008). Recovery capital as prospective predictor of sustained recovery, life satisfaction, and stress among former poly-substance users. Substance Use and Misuse, 43(1), 27–54. 10.1080/10826080701681473 18189204 PMC2211734

[bibr57-10497323231174161] Leamy M. Bird V. le Boutillier C. Williams J. Slade M. (2011). Conceptual framework for personal recovery in mental health: Systematic review and narrative synthesis. The British Journal of Psychiatry: The Journal of Mental Science, 199(6), 445–452. 10.1192/bjp.bp.110.083733 22130746

[bibr58-10497323231174161] Liebregts N. van der Pol P. de Graaf R. van Laar M. van den Brink W. Korf D. J. (2015). Persistence and desistance in heavy cannabis use: The role of identity, agency, and life events. Journal of Youth Studies, 18(5), 617–633. 10.1080/13676261.2014.992320

[bibr59-10497323231174161] Lindgren K. P. Neighbors C. Gasser M. L. Ramirez J. J. Cvencek D. (2017). A review of implicit and explicit substance self-concept as a predictor of alcohol and tobacco use and misuse. The American Journal of Drug and Alcohol Abuse, 43(3), 237–246. 10.1080/00952990.2016.1229324 27715328 PMC5384879

[bibr60-10497323231174161] Lock C. A. (2004). Alcohol and brief intervention in primary health care: What do patients think? Primary Health Care Research and Development, 5(2), 162–178. 10.1191/1463423604pc194oa

[bibr61-10497323231174161] Mackintosh V. Knight T. (2012). The notion of self in the journey back from addiction. Qualitative Health Research, 22(8), 1094–1101. 10.1177/1049732312450325 22707345

[bibr62-10497323231174161] Martinelli T. F. Nagelhout G. E. Bellaert L. Best D. Vanderplasschen W. van de Mheen D. (2020). Comparing three stages of addiction recovery: Long-term recovery and its relation to housing problems, crime, occupation situation, and substance use. Drugs: Education, Prevention and Policy, 27(5), 387–396. 10.1080/09687637.2020.1779182

[bibr63-10497323231174161] Martinelli T. F. vander Laenen F. Nagelhout G. E. van de Mheen D. H. (2022). Addiction and recovery in Dutch governmental and practice-level drug policy: What’s the problem represented to be? Journal of Drug Issues, 52(4), 547–567. 10.1177/00220426221087590

[bibr64-10497323231174161] Maslow A. H. (1943). A theory of human motivation. Psychological Review, 50(4), 370–396. 10.1037/h0054346

[bibr65-10497323231174161] McIntosh J. McKeganey N. (2000). Addicts’ narratives of recovery from drug use: Constructing a non-addict identity. Social Science and Medicine, 50(10), 1501–1510. 10.1016/S0277-9536(99)00409-8 10741584

[bibr66-10497323231174161] McLellan A. T. Lewis D. C. O’Brien C. P. Kleber H. D. (2000). Drug dependence, a chronic medical illness: Implications for treatment, insurance, and outcomes evaluation. JAMA, 284(13), 1689–1695. 10.1001/jama.284.13.1689 11015800

[bibr67-10497323231174161] Mellor R. Lancaster K. Ritter A. (2020) Recovery from alcohol problems in the absence of treatment: A qualitative narrative analysis. Addiction. 116(6), 1413–1423. 10.1111/add.15288 33037842

[bibr68-10497323231174161] Monico L. B. Ludwig A. Lertch E. Mitchell S. G. (2020). Using timeline methodology to visualize treatment trajectories of youth and young adults following inpatient opioid treatment. International Journal of Qualitative Methods, 19, 160940692097010. 10.1177/1609406920970106

[bibr69-10497323231174161] Moos R. H. Moos B. S. (2007). Protective resources and long-term recovery from alcohol use disorders. Drug and Alcohol Dependence, 86(1), 46–54. 10.1016/j.drugalcdep.2006.04.015 16769181

[bibr70-10497323231174161] Neale J. Hons B. A. Ma C. Neale J. (1998). Drug users' views of drug service providers. Health and Social Care in the Community, 6(5), 308–317. 10.1046/j.1365-2524.1998.00140.x 11560602

[bibr71-10497323231174161] NIDA . (2020). Drug misuse and addiction. National Institute on Drug Abuse. https://www.drugabuse.gov/publications/drugs-brains-behavior-science-addiction/drug-misuse-addiction

[bibr72-10497323231174161] Patton D. Best D. Brown L. (2022). Overcoming the pains of recovery: The management of negative recovery capital during addiction recovery pathways. Addiction Research and Theory, 30(5), 340–350. 10.1080/16066359.2022.2039912

[bibr73-10497323231174161] Roy A. Galvani S. Clayson A. (2022). Recovery as long term: An introduction. In Long-term recovery from substance use (pp. 3–14). Policy Press. 10.51952/9781447358190.ch001

[bibr74-10497323231174161] Scott C. K. Dennis M. L. Grella C. E. Watson D. P. (2021). Improving retention across the OUD service cascade upon reentry from jail using Recovery Management Checkups-Adaptive (RMC-A) experiment. Journal of Substance Abuse Treatment, 128, 108245. 10.1016/j.jsat.2020.108245 33461829 PMC8192586

[bibr75-10497323231174161] Sexton Topper P. Bauermeister J. A. (2021). Relationship timelines, dyadic interviews, and visual representations: Implementation of an adapted visual qualitative technique. International Journal of Qualitative Methods, 20, 160940692110167. 10.1177/16094069211016708

[bibr76-10497323231174161] Simpson D. D. Joe G. W. (2004). A longitudinal evaluation of treatment engagement and recovery stages. Journal of Substance Abuse Treatment, 27(2), 89–97. 10.1016/j.jsat.2004.03.001 15450643

[bibr89-10497323231174161] Simpson . 10.1186/s13011-020-0254-x

[bibr1000-10497323231174161] Svendsen . 10.1186/s13011-020-0254-x

[bibr77-10497323231174161] Thom B. Brown C. Drummond C. Edwards G. Mullan M. (1992). The use of services for alcohol problems: General practitioner and specialist alcohol clinic. British Journal of Addiction, 87(4), 613–624. 10.1111/j.1360-0443.1992.tb01963.x 1317237

[bibr78-10497323231174161] Vaillant G. E. (2003). A 60-year follow-up of alcoholic men. Addiction, 98(8), 1043–1051. 10.1046/j.1360-0443.2003.00422.x 12873238

[bibr79-10497323231174161] van der Stel J. (2013). Innovatie rond herstel. Verslaving, 9(4), 5–18. 10.1007/s12501-013-0032-9

[bibr80-10497323231174161] van der Stel J. (2020). Herstel als leerproces. SWP.

[bibr81-10497323231174161] van Steenberghe T. Vanderplasschen W. Bellaert L. de Maeyer J. (2021). Photovoicing interconnected sources of recovery capital of women with a drug use history. Drugs: Education, Prevention and Policy, 28(5), 411–425. 10.1080/09687637.2021.1931033

[bibr82-10497323231174161] van Weeghel J. van Zelst C. Boertien D. Hasson-Ohayon I. (2019). Conceptualizations, assessments, and implications of personal recovery in mental illness: A scoping review of systematic reviews and meta-analyses. Psychiatric Rehabilitation Journal, 42(2), 169–181. 10.1037/prj0000356 30843721

[bibr83-10497323231174161] Waldorf D. Biernacki P. (1981). The natural recovery from opiate addiction: Some preliminary findings. Journal of Drug Issues, 11(1), 61–74. 10.1177/002204268101100104

[bibr84-10497323231174161] White W. L. (2012). Recovery/Remission from Substance Use Disorders: An analysis of reported outcomes in 415 scientific reports. The Philadelphia Department of Behavioral Health and Intellectual disability Services and the Great Lakes Addiction Technology Transfer Center. https://www.naadac.org/assets/1959/whitewl2012_recoveryremission_from_substance_abuse_disorders.pdf

[bibr85-10497323231174161] Whitley R. Crawford M. (2005). Qualitative research in psychiatry. The Canadian Journal of Psychiatry, 50(2), 108–114. 10.1177/070674370505000206 15807227

[bibr86-10497323231174161] Wiers R. W. Rinck M. Kordts R. Houben K. Strack F. (2010). Retraining automatic action-tendencies to approach alcohol in hazardous drinkers. Addiction, 105(2), 279–287. 10.1111/j.1360-0443.2009.02775.x 20078486

[bibr87-10497323231174161] Winick C. (1962). Maturing out of narcotic addiction. Bulletin on Narcotics, 14(1), 1–7.

