# Supplementary material for: Understanding the Process of Drug Addiction Recovery Through First-Hand Experiences: A Qualitative Study in the Netherlands Using Lifeline Interviews
Source: Qual Health Res. 2023 Jun 6;33(10):857–70. doi: 10.1177/10497323231174161 (PMC10426251; doi:10.1177/10497323231174161)
Supplement: Supplemental Material - Understanding the Process of Drug Addiction Recovery Through First-Hand Experiences: A Qualitative Study in the Netherlands Using Lifeline Interviews [file sj-pdf-1-qhr-10.1177_10497323231174161.pdf]

Supplementary table 1. Thematic table connecting themes, sub-themes and codes

Theme	Sub-themes	Codes	Examples of initial codes
**1: Recovery is a broad process of change because addiction is interwoven with everything**	Recovery is more than not using drugs	Being abstinent is not the same as being in recovery	Not using drugs did not improve situation
			Experiencing problems while in recovery
			Changed environment or moved for recovery
		Addiction is also in other behavior	Problems with sexual behavior and relations while in addiction
			Mental health problems linked to addiction
			Doing 'good' as part of recovery
	Broad recovery goals	Having practical goals was important	Achieving a day routine
			Achieving financial stability
			Acquiring a job
		Expecting emotional gains	Trying to become happier
			Trying to achieve serenity
**2: Recovery is reconsidering identity, seeing things in a new light**		Gaining control	Considering and taking care of own needs
			Staying close to myself
			Getting to know myself
		Identity change	Adopt non-user identity
			Dealing with vulnerabilities
		Seeing things in a new light	Diagnosis of behavior
			Is controlled use possible for me?
			Learning about self through helping others
**3: Recovery is a staged long-term process**	Planting a seed	Past events facilitated change	Emotionally touched by an encounter
			Life impacting events
			Previous recovery attempts helped later attempt
	Stages of recovery	Turning points	Period to stabilize
			Intense focus on recovery
			Maintaining recovery
			Broadening recovery goals
		Changing meaning of recovery	Broadening recovery goals
			Finding new sources of motivation
			Being able to do new things
	Recovering or recovered?	Finding new things to recover	Broadening recovery goals
		Moving on	Detaching from addiction / recovery identity
		Becoming rebellious or stubborn in recovery	Finding an own way to maintain recovery
**4: Universal life processes are part of recovery**		Normal or universal processes	Becoming older
			Changing lifestyle preferences
			Not so different from people without addiction experiences
		Insights from recovery apply to other things	People without addiction experiences benefit from recovery insights or experiences
			This should be taught at schools
			Everyone needs a bit of recovery
